# Impact of Social Distancing Measures on Coronavirus Disease Healthcare Demand, Central Texas, USA

**DOI:** 10.3201/eid2610.201702

**Published:** 2020-10

**Authors:** Xutong Wang, Remy F. Pasco, Zhanwei Du, Michaela Petty, Spencer J. Fox, Alison P. Galvani, Michael Pignone, S. Claiborne Johnston, Lauren Ancel Meyers

**Affiliations:** The University of Texas at Austin, Austin, Texas, USA (X. Wang, R.F Pasco, Z. Du, M. Petty, S.J. Fox, L. Ancel Meyers);; Yale University School of Public Health, New Haven, Connecticut, USA (A.P. Galvani);; The University of Texas at Austin Dell Medical School, Austin (M. Pignone, S. Claiborne Johnston);; Santa Fe Institute, Santa Fe, New Mexico, USA (L. Ancel Meyers)

**Keywords:** coronavirus disease, COVID-19, severe acute respiratory syndrome coronavirus 2, SARS-CoV-2, coronavirus, viruses, epidemiology, pandemics, mathematical model, hospital bed capacity, social distancing, measures, healthcare demand, respiratory infections, zoonoses, Texas, United States

## Abstract

Social distancing orders have been enacted worldwide to slow the coronavirus disease (COVID-19) pandemic, reduce strain on healthcare systems, and prevent deaths. To estimate the impact of the timing and intensity of such measures, we built a mathematical model of COVID-19 transmission that incorporates age-stratified risks and contact patterns and projects numbers of hospitalizations, patients in intensive care units, ventilator needs, and deaths within US cities. Focusing on the Austin metropolitan area of Texas, we found that immediate and extensive social distancing measures were required to ensure that COVID-19 cases did not exceed local hospital capacity by early May 2020. School closures alone hardly changed the epidemic curve. A 2-week delay in implementation was projected to accelerate the timing of peak healthcare needs by 4 weeks and cause a bed shortage in intensive care units. This analysis informed the Stay Home-Work Safe order enacted by Austin on March 24, 2020.

Severe acute respiratory syndrome coronavirus 2 (SARS-CoV-2) appeared in Wuhan, China, during December 2019, and coronavirus disease (COVID-19) caused by this virus was declared a pandemic on March 11, 2020, by the World Health Organization (*1*). As of June 24, a total of 193 countries, areas, or territories had reported 9,129,146 confirmed COVID-19 cases and 473,797 deaths. Substantial outbreaks have occurred in India, Russia, Brazil, and the United States; the United States has the highest cumulative confirmed number of cases and deaths (*2*).

The United States reported its first imported SARS-CoV-2 case from Wuhan on January 20, in Washington (*3*), 6 days ahead of California (*4*) and 40 days ahead of New York, New York (*5*); the first locally infected cases were reported on February 28 (*6*). As of June 24, all 50 states had reported confirmed cases, 48 had reported community spread, and cumulative confirmed COVID-19 cases were 2,336,615 and deaths were 121,117 (*7*). Surges in COVID-19 hospitalizations have compromised local healthcare systems in New York (*8*) and Seattle (*9*).

Beginning in March 2020, states and cities implemented extensive social distancing measures to contain the spread of SARS-CoV-2, including school closures, limits on mass gatherings, shelter-in-place orders, travel restrictions, and bans on nonessential commercial activities. By early April, 45 states had issued a statewide shelter-in-place order or >1 city-level stay-at-home order, affecting >316 million persons. As of June 25, all measures have expired or relaxed (*10*). The timing of the orders varied; California was the first state to enact strict orders on March 19 and South Carolina the last on April 7 (*10*). These measures dramatically slowed the pace of the pandemic during April and May, but confirmed COVID-19 cases and hospitalizations have been increasing since early June, particularly in Arizona, Florida, Texas, and California (*11*).

As COVID-19 emerged into a global threat, we took a national pandemic influenza model that was built through a pandemic preparedness contract with the Centers for Disease Control and Prevention (CDC; Atlanta, GA, USA) and adapted it to model the spread and control of COVID-19 within and between 217 US cities. We used this model to project the potential effects of school closures coupled with social distancing, in terms of reducing cases, deaths, hospitalizations, intensive care unit (ICU) visits, and ventilator needs, on local, regional, and national scales. We have focused our analysis on Austin, which is the capital of Texas and the fastest growing city in the United States, as a representation of major US metropolitan areas. The scenarios and inputs (e.g., epidemiologic parameters) were determined in consultation with CDC and the Regional Healthcare System Executive Council of the Austin–Travis County Emergency Operations Command.

## Methods

We focused on the Austin–Round Rock Statistical Metropolitan Area, which had a population of 2.17 million persons in 2018, but the qualitative findings and impact of social distancing will apply to cities throughout the United States. We analyzed a compartmental model that incorporates age-specific high risk proportions and contact rates to measure the effects of 2 key interventions, school closures and social distancing measures, which reduce nonhousehold contacts by a specified percentage. We estimated the effects of these measures on cases, hospitalizations, ICU visits, ventilator needs, and deaths.

We built a stochastic age- and risk-structured susceptible-exposed-asymptomatic-symptomatic-hospitalized-recovered (SEAYHR) model of SARS-CoV-2 transmission (Appendix Figure 1). Persons were separated into 5 age groups, <1–4, 5–17, 18–49, 50–64, and >65 years of age, on the basis of population data for the 5-county Austin–Round Rock Metropolitan Area from the 2017 American Community Survey (*12*). Each age group was divided into a low-risk and high-risk group on the basis of prevalence of chronic conditions estimated for the Austin population (Appendix Figure 2) (*13*–*16*). We also estimated the proportion of pregnant women in each age group as a special risk class (*17*). All persons were assumed to be susceptible to the disease. Infected persons were modeled to enter a latent period in which they were symptom-free and not yet infectious (*18*) and then progressed to either a symptomatic or asymptomatic compartment, both infectious. Asymptomatic persons were assumed to have the same infectious period as symptomatic persons but lower infectiousness. The rates at which symptomatic case-patients were moved to a hospitalized compartment and died depended on age and risk group. Recovered persons were considered fully immune. Deaths were assumed to occur after hospitalization. We provide a detailed description of the methods used (Appendix).

**Figure 1:**
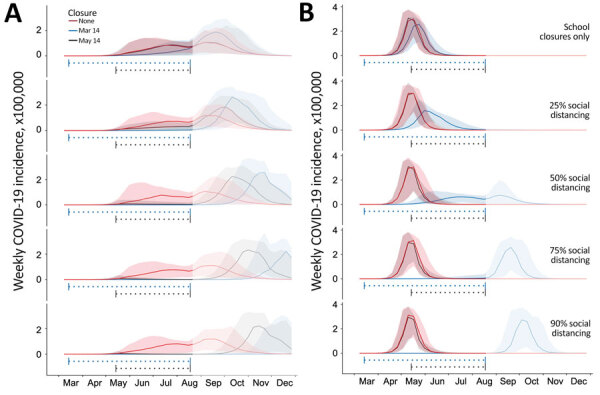
Projected weekly incident of COVID-19 cases in Austin–Round Rock Metropolitan Statistical Area, Texas, USA. Graphs show simulation results for different levels of social distancing and implementation times, assuming an epidemic doubling time of A) 7.2 days (*18*–*20**,**22*) or B) 4 days (*22*–*24*). Each graph displays 3 projections: a baseline assuming no social distancing (red), social distancing implemented March 14–Aug 17, 2020 (blue), and social distancing implemented May 14–Aug 17, 2020 (black). From top to bottom, the graphs in each column correspond to increasingly stringent social distancing measures: school closures plus social distancing that reduces nonhousehold contacts by 0%, 25%, 50%, 75%, or 90%. Solid lines indicate medians of 100 stochastic simulations; shading indicates inner 95% ranges of values. The horizontal dotted lines beneath the curves indicate intervention periods. The faded mid-August to December time range indicates long-range uncertainty regarding COVID-19 transmission dynamics and intervention policies. COVID-19, coronavirus disease.

**Figure 2:**
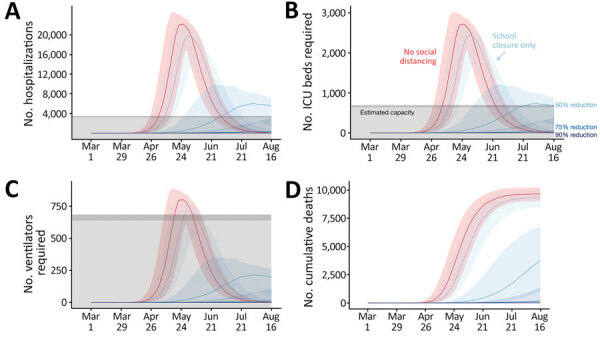
Projected COVID-19 healthcare demand and cumulative deaths in Austin–Round Rock Metropolitan Statistical Area, Texas, USA. Graphs show simulation results across multiple levels of social distancing, assuming a basic reproductive number of 2.2 with a 4-day epidemic doubling time. Extensive social distancing is expected to substantially reduce the burden of COVID-19 A) hospitalizations, B) patients requiring ICU care, C) patients requiring mechanical ventilation, and D) cumulative deaths. Red lines indicate projected COVID-19 transmission assuming no interventions under the parameters given in Table A1. Blue lines indicate increasing levels of social distancing interventions, from light to dark: school closures plus social distancing interventions that reduce nonhousehold contacts by either 0%, 50%, 75%, or 90%. Lines and shading indicate medians and inner 95% ranges of values across 100 stochastic simulations. Gray shaded region indicates estimated surge capacity for COVID-19 patients in the Austin-Round Rock Metropolitan Statistical Area as of March 28, 2020, which is calculated on the basis of 80% of the total 4,299 hospital beds, 90% of the total 755 ICU beds, and 755 mechanical ventilators. COVID-19, coronavirus disease; ICU, intensive care unit.

All model parameters (Appendix Tables 1–3) were based on published estimates from COVID-19 studies, as well as input from CDC and Austin. We assumed a basic reproduction number (R_0_) of 2.2 (*19*) and considered 2 different doubling times, 7.2 days (low growth rate) (*19**–**21*) and 4 days (high growth rate) (*22*–*24*). We provide a sensitivity analysis for an R_0_ of 3.5 (Appendix). Age-specific contact rates were estimated by using contact matrices published by Prem et al. and are adjusted to model school closures and various levels of social distancing (*25*). Transmission rates were estimated by fitting simulations to a given R_0_ and epidemic doubling time. The latent period (i.e., noninfectious beginning of the incubation period) was sampled from a triangular distribution from 1.9 days to 3.9 days and a mean of 2.9 days (*26*,*27*), and the infectious period was sampled from a triangular distribution from 5.3 days to 7.3 days and a mean of 6.3 days (*27*). We assumed that 43% of infections are asymptomatic and that asymptomatic cases are two thirds as infectious as symptomatic cases (*28*,*29*). Following planning scenarios of CDC, we assumed that the infection hospitalization rate and infection fatality rate was 10 times higher in high-risk than low-risk persons within each age group.

Simulations began with 5 imported symptomatic cases in the 18–49-year-old age group on March 1, 2020, and were updated at 2.4-hour intervals. For each combination of epidemic scenarios (low/high growth rate) and intervention strategies (school closure policy with different levels of social distancing), we ran 100 stochastic simulations and reported the medians and 95% prediction intervals (ranges) at weekly intervals.

### School Closure Policies

As part of a CDC modeling network, we initially modeled a large number of school closure policies, with variable implementation time and duration. To simulate school closures, we decreased the daily age-specific contact rates by the estimated number of contacts that occur within schools (*25*,*30*). The school-specific contact numbers encompass all interactions among students and teachers occurring at all educational levels, from elementary schools through colleges and universities. In our model, school closures reduced daily contacts by 15% for persons <1–4 years of age, 26% for persons 5–17 years of age, 9% for persons 18–49 of age, 9% for persons 50–64 of age, and 2% for persons >64 years of age. We reported only 2 of these strategies to demonstrate the effects of implementation time: closure immediately after the first confirmed case (March 14) and delayed closure 2 months after the first confirmed case (May 14). In both cases, we assumed that schools remain closed through the end of the summer vacation (August 18, 2020), which corresponds to a 23-week duration for the early closure and a 14-week duration for the late closure. The early closure scenario roughly corresponded to Austin announcing the first 2 confirmed cases on March 13 and major school districts closing the next day. In our simulations, the median cumulative number of symptomatic COVID-19 cases by March 14th was 38 (interquartile range [IQR] 27–53) and 14 (IQR 9–19), assuming a 4-day and 7-day doubling period, respectively; by May 14, median cumulative symptomatic cases increased to 530,426 (IQR 114,151–783,667) and 3,206 (IQR 561–7,611), respectively.

### Social Distancing Measures

In addition to school closures, we considered the effect of various levels of social distancing that decreased nonhousehold contacts by 25%, 50%, 75%, and 90% overall. These levels were chosen to correspond to increasingly more severe levels of restriction on social interaction from limiting large crowds to near-total restriction on out-of-home movement except for healthcare and basic necessities.

Age-stratified contact rates (*25*) were derived from the POLYMOD diary-based study in Europe (*30*) and separated in contacts occurring at home, at school, at work and elsewhere. We used the national US age distribution (*31*) to aggregate these estimates from 17 to the 5 age groups of our model (Appendix Tables 4–7). We combined these matrices to model 4 different types of days: normal school days (all contacts); normal weekends and short weekday holidays (all but school and work contacts; adults are assumed to work during the long summer break); weekdays during school closures/social distancing; and weekend or weekday holiday during school closure/social distancing. To model school closures with social distancing, we included all household contacts plus a specified proportion of contacts outside the home. On weekdays, this proportion included a proportion of contacts occurring at work and elsewhere; on weekends and holidays (excluding summer vacation), it included just contacts occurring elsewhere. Days were assigned to 1 of these 4 contact models on the basis of the 2019–2020 and 2020–2021 school calendars from the Austin Independent School District, which was the largest public school district in the metropolitan area, serving ≈22.7% of the Austin–Round Rock Statistical Metropolitan Area population.

### Healthcare Demands

We assumed that hospitalized cases were admitted on average 5.9 days (L. Tindale et al., unpub. data, https://doi.org/10.1101/2020.03.03.20029983) after symptom onset, with the infection hospitalization rate depending on the age and risk group (Appendix Table 1). Hospitalized case-patients who recovered were considered discharged an average of 11 days after admission; deaths occurred an average of 7.82 days after admission. We estimated the number ICU beds and ventilators needed to care for COVID-19 case-patients each day on the basis of age-specific rates provided by the CDC (Appendix Table 3) and assuming that the average duration of ICU care and ventilation support are 8 days and 5 days, respectively. There is some uncertainty regarding how these estimates might change when healthcare facilities reach or exceed capacity because of a lack of available postdischarge care and inefficiency in the healthcare system caused by worker illness. Thus, we also tested an alternative scenario with longer duration of hospital stay, ICU care, and ventilation (Appendix). We did not consider potential excess deaths resulting from lack of access to adequate healthcare during pandemic surges.

## Results

Our analyses focus on 2 key levers of intervention: the speed of implementation and the extent of social distancing. We considered 2 scenarios for the epidemic growth rate of COVID-19 and project 5 outcomes: cases, hospitalizations, ICU care, ventilator needs, and deaths.

Regardless of epidemic growth rate, school closures alone had little effect on the burden of the epidemic. These closures would flatten the curve slightly if enacted immediately after the detection of the first case (Figure 1). High levels of social distancing, when coupled with school closures, substantially delayed and dampened the epidemic peak. The impact of the measures depended on early implementation. Under both the slower and faster epidemic growth scenarios (i.e., 7-day and 4-day doubling times), immediate measures beginning on March 14 were much more effective than 2-month delayed measures at slowing transmission throughout the spring and summer of 2020 (Figure 1). Given that recent estimates for the doubling time in US cities are short, ranging from 2.4 to 3 days (*24*,*32*), this finding suggests that proactive measures were justified, because delayed measures would have been almost entirely ineffective. If the reproduction is higher than we assumed, then more vigilant social distancing would be required to slow spread (Appendix). Although the immediate school closure on March 14 had little impact on the initial wave, the August opening of schools would be expected to amplify a fall wave if the population is not yet close to herd immunity.

To assess the impact of social distancing measures on mitigating healthcare surge in the Austin–Round Rock Statistical Metropolitan Area, we considered the more plausible 4-day doubling time scenario (Table 1; Figure 2). Social distancing measures that reduced nonhousehold contacts by <50% were projected to delay but not prevent a healthcare crisis. Only the 75% and 90% contact reduction scenarios were projected to reduce hospitalizations, ICU care, and ventilator needs below the estimated capacity for the metropolitan area (Table 2). If 50% social distancing were implemented on March 28 instead of March 14 (i.e., a 2-week delay), we would expect COVID-19 ICU requirements to exceed local capacity by the end of June instead of only reaching capacity by the end of July (i.e., a 4-week acceleration) (Appendix). Under scenarios that predict overwhelming healthcare surges, we likely underestimate deaths because we do not account for excess deaths for persons with COVID-19 or other medical conditions, such as cancer or cardiovascular disease, who might not receive timely or safe care.

**Table 1:** Estimated cumulative COVID-19 cases, healthcare requirements, and deaths, Austin–Round Rock, metropolitan statistical area, Texas, USA, March 1–August 17, 2020*

Outcome	No measures	School closure	School closure and 50% social distancing	School closure and 75% social distancing	School closure and 90% social distancing
Cases	1,139,633 (1,092,754–1,173,408)	1,098,755 (1,016,794–1,143,147)	596,304 (215,897–854,094)	34,232 (2,871–244,959)	2,013 (642–11,358)
Hospitalizations	79,120 (75,373–82,608)	76,698 (70,091–80,602)	36,534 (11,474–57,912)	1,889 (159–13,512)	125 (32–660)
ICU	13,312 (12,673–13,890)	12,897 (11,786–13,540)	6141 (1,929–9,736)	318 (27–2,273)	21 (5–111)
Ventilators	6,274 (5,973–6,545)	6,077 (5,554–6,377)	2,893 (909–4,587)	150 (13–1,071)	10 (3–53)
Deaths	9,646 (9,031–10,206)	9,324 (8,481–9,954)	3,698 (995–6,751)	176 (13–1,315)	13 (1–70)

*Values are medians (95% prediction intervals) across 100 stochastic simulations based on parameters in Table 1. COVID-19, coronavirus disease; ICU, intensive care unit.

**Table 2:** Estimated peak COVID-19 cases and healthcare demands, Austin–Round Rock, metropolitan statistical area, Texas, USA, March 1–August 17, 2020*

Outcome	No measures	School closure	School closure and 50% social distancing	School closure and 75% social distancing	School closure and 90% social distancing
Cases	272,978 (228,088–327,181)	237,428 (176,910–281,441)	64,779 (33,837–110,968)	4,643 (267–35,148)	163 (42–1,279)
New cases daily	54,106 (47,301–62,646)	43,535 (33,691–50,105)	10,573 (6,297–16,768)	851 (57–5,436)	32 (10–212)
Hospitalizations	23,073 (20,961–24,695)	20,671 (17,193–22,473)	6,804 (3,088–10,271)	402 (31–2,963)	18 (5–105)
ICU	2,831 (2,575–3,033)	2,532 (2,107–2,759)	833 (377–1,254)	50 (4–362)	2 (1–13)
Ventilators	835 (760–895)	746 (621–814)	245 (111–369)	15 (1–107)	1 (0–4)

*Values are medians (95% prediction intervals) across 100 stochastic simulations based on parameters in Table 1. COVID-19, coronavirus disease; ICU, intensive care unit.

Under the naive scenario that school closures and social distancing measures are lifted entirely on the first day of the 2020–2021 academic year (August 18) (*33*), the pace and extent of COVID-19 transmission in the fall would depend on how many persons were infected (and thereby immunized) during the spring and summer (Figure 1). As cumulative incidence approaches the herd immunity threshold of roughly 55% of the population, the effective reproduction number (R_t_) decreases. Once this 55% threshold is surpassed, the reproduction number decreases below 1, and the virus would be unable to spread widely, even if social distancing measures are lifted. Assuming the faster 4-day epidemic doubling time (Figure 1, panel B), a minimum of 50% social distancing is necessary to suppress transmission over the summer. Under 75% or 90% social distancing, the lifting of measures on August 18 would be expected to produce epidemic peaks in the middle or end of September, respectively. Assuming the slower 7-day doubling time (Figure 1, panel A), even delayed social distancing would be expected to forestall the start of the epidemic from spring to fall. The higher fall peaks that were produced under the most extreme social distancing, assuming a 7-day doubling time, stem from baseline contact patterns (in the absence of social distancing): a COVID-19 epidemic that begins in the spring would be naturally dampened by the 3-month summer vacation period when children are out of school, whereas a fall start would be amplified by the start of the academic year.

## Discussion

As COVID-19 emerged as a global threat in early 2020, we rapidly adapted a pandemic influenza model that was under development as part of an effort coordinated by CDC to build a strategic national modeling resource for pandemic planning and response. The analyses provided in this report originated in time-sensitive requests from CDC, Austin, and the state of Texas to evaluate the potential impact of school closures and social distancing on the emergence and spread of COVID-19 in US cities. Our projections indicate that, without extensive social distancing measures, the emerging outbreak would quickly surpass healthcare capacity in the region. However, with extensive social distancing, the number of cases, hospitalizations, and deaths could be substantially reduced throughout the summer of 2020. Although these analyses are specific to the Austin–Round Rock metropolitan area, we expect that the impacts of the mitigation strategies will be qualitatively similar for cities throughout the United States.

Our epidemiologic projections and conclusions regarding the urgent need for extensive social distancing are consistent with a recent analysis by Imperial College London (*34*). However, we assume that a lower percentage of hospitalized patients receive critical care (15%–20% vs. 30%) and consequently project a lower peak ICU demand. In sensitivity analyses with more extreme assumptions about critical care requirements, the projected peak demand increases accordingly (Appendix). The local focus of our model, which incorporates city-specific data regarding demographics, high-risk conditions, contact patterns, and healthcare resource availability, enables us to project near-term healthcare demands and provide actionable insights for local healthcare and governmental decision-makers.

We conducted these analyses to inform decision making in a rapidly evolving environment with substantial uncertainty. On March 6, 2020, Austin declared a local state of disaster and cancelled the South by Southwest Conference and Festival, which was expected to draw 417,400 visitors from around the world and bring $355.9 million to the local economy (*35*). Evidence of community transmission appeared within days of the first confirmed COVID-19 case in Austin on March 13. Shortly after, the University of Texas at Austin, one of the largest public universities with >50,000 students, and the largest public school district in Austin announced school closures (*36*,*37*). On March 24, Austin issued a Stay Home-Work Safe order to eliminate all nonessential business and travels (*38*). Leaders of Austin requested the healthcare analyses (Figure 2) in the days leading up to the order of March 24 and requested that we release a preliminary report to educate the public (*39*).

Social distancing measures, including school closures, restrictions on travel, mass gatherings and commercial activities, and more extensive shelter-in-place advisories, aim to decrease disease transmission within a population by preventing contacts between persons. Our analyses project the effect of such measures on the transmission dynamics of COVID-19 but do not consider the economic, social and psychological costs of social distancing measures, including the socioeconomic disparities in burden and illness and death resulting from reductions in health and mental healthcare services (*40*,*41*).

There is an urgent need to project the relative effects of different levels of social distancing in light of their potential societal costs, including school closures, partial work and travel restrictions and cocooning of the high risk, so that restrictions can be strategically lifted without compromising public health. In particular, school closures are often deployed earlier than more extensive social distancing measures. However, such closures can be costly, particularly for low-income families who might rely on lunch programs and be unable to afford childcare (*42*,*43*), and our analysis suggests that they might only slightly reduce the pace of transmission and peak hospital surge. However, the role of children in community transmission of COVID-19 remains uncertain; thus, school closures are prudent at this time. Children represent a low proportion of confirmed cases worldwide (*44*,*45*), perhaps reflecting that COVID-19 is less severe in children than adults (*46*). If we learn that the prevalence or infectiousness of COVID-19 is low in children, then opening schools may be a reasonable first step toward resuming normalcy.

Although our model incorporates considerable detail regarding the natural history of COVID-19, age- and location-specific contact patterns, and the demographic and risk composition of the Austin–Round Rock Statistical Metropolitan Area, it does not explicitly capture neighborhood, household, or other community structure that can serve to amplify or impede transmission (*47*–*49*). In addition, we ignored the possible importation of COVID-19 cases from other cities, under the assumption that the additional cases would have a negligible impact, particularly during the period of exponential growth. Although large numbers of introductions could undermine mitigation efforts that radically suppress transmission, we conjecture that such efforts would include travel restrictions, contact tracing and other measures to contain emerging clusters. Our model also does not evaluate other potentially effective interventions, such as increased levels of selective testing and isolation.

These analyses rely on recently published estimates for transmission rate and severity of COVID-19, as well as best estimates from expert opinions from CDC and Dell Medical School. There is still much we do not understand about the transmission dynamics of SARS-CoV-2, including its R_0_, the infectiousness of asymptomatic case-patients (*28*), and the extent to which infections confer future immunity (*50*), all of which are key to anticipating future pandemic waves. As of June 2020, it is likely most US cities remain far from herd immunity. Even in New York, New York, which experienced a substantially larger first wave than other cities, serologic surveys suggest that only 22.7% of the population has been exposed (E.S. Rosenberg et al., unpub. data, https://doi.org/10.1101/2020.05.25.20113050). However, summer surges in transmission in some cities might infect large numbers of persons by the beginning of the fall semester. In that case, resolving these key uncertainties will be critical to projecting the full impact of school openings. Our understanding of COVID-19 is evolving so rapidly that we expect there might be consensus around different estimates for key transmission and severity parameters by the time this work is published. Thus, we emphasize the qualitative but not quantitative results of the analysis.

Given the rapid spread of COVID-19, early and extensive social distancing are both viable and necessary for preventing catastrophic hospital surges. Despite this study’s uncertainties in key parameters and the focus on a single city, the expansion and containment of COVID-19 in cities worldwide suggest that these insights are widely applicable. This framework can be updated as situational awareness of COVID-19 improves to provide a quantitative sounding board as public health agencies evaluate strategies for mitigating risks while sustaining economic activity in the United States.

Appendix Additional information on impact of social distancing measures on coronavirus disease healthcare demand, central Texas, USA.
