# Supplementary material for: Impact of Social Distancing Measures on Coronavirus Disease Healthcare Demand, Central Texas, USA
Source: Emerg Infect Dis. 2020 Oct;26(10):2361–9. doi: 10.3201/eid2610.201702 (PMC7510701; doi:10.3201/eid2610.201702)
Supplement: Appendix — Additional information on impact of social distancing measures on coronavirus disease healthcare demand, central Texas, USA. [file 20-1702-Techapp-s1.pdf]

Article DOI: https://doi.org/10.3201/eid2510.201702

Impact of Social Distancing Measures on Coronavirus Disease Healthcare Demand, Central Texas, USA

Appendix.

Section 1. COVID-19 Epidemic Model Structure and Parameters

The model structure is diagrammed in Appendix Figure 1 and described in the equations below.

For each age and risk group, we build a separate set of compartments to model the transitions between the states: susceptible (S), exposed (E), symptomatic infectious (I^Y), asymptomatic infectious (I^A), symptomatic infectious that are hospitalized (I^H), recovered (R), and deceased (D). The symbols S, E, I^Y, I^A, I^H, R, and D denote the number of people in that state in the given age/risk group and the total size of the age/risk group is $N = S + E + I^Y + I^A + I^H + R + D$.

The model for individuals in age group a and risk group r is given by:

$$\frac{dS_{a,r}}{dt} = -\sum_{i\in A}\sum_{j\in K}\left(I^Y_{i,j}\omega^Y + I^A_{i,j}\omega^A + E_{i,j}\omega^E\right)\beta\phi_{a,i}/N_i$$

$$\frac{dE_{a,r}}{dt} = \sum_{i\in A}\sum_{j\in K}\left(I^Y_{i,j}\omega^Y + I^A_{i,j}\omega^A + E_{i,j}\omega^E\right)\beta\phi_{a,i}/N_i - \sigma E_{a,r}$$

$$\frac{dI^A_{a,r}}{dt} = (1-\tau)\,\sigma E_{a,r} - \gamma^A I^A_{a,r}$$

$$\frac{dI^Y_{a,r}}{dt} = \tau\sigma E_{a,r} - (1-\pi)\,\gamma^Y I^Y_{a,r} - \pi\eta I^Y_{a,r}$$

$$\frac{dI^H_{a,r}}{dt} = \pi\eta I^Y_{a,r} - (1-\nu)\,\gamma^H I^H_{a,r} - \nu\mu I^H_{a,r}$$

$$\frac{dR_{a,r}}{dt} = \gamma^A I^A_{a,r} + (1-\pi)\,\gamma^Y I^Y_{a,r} + (1-\nu)\,\gamma^H I^H_{a,r}$$

$$\frac{dD_{a,r}}{dt} = \nu\mu I^H_{a,r}$$

where A and K are all possible age and risk groups, ω^A, ω^Y, ω^H are relative infectiousness of the I^A, I^Y, E compartments, respectively, β is transmission rate, $\phi_{a,i}$ is the mixing rate between age group a, $i \in A$, γ^A,γ^Y, γ^H are the recovery rates for the I^A, I^Y, I^H compartments, respectively, σ is the exposed rate, τ is the symptomatic ratio, π is the proportion of symptomatic individuals requiring hospitalization, η is rate at which hospitalized cases enter the hospital following symptom onset, ν is mortality rate for hospitalized cases, and μ is rate at which terminal patients die.

Initial conditions, school closures and social distancing policies are shown in Appendix Table 1. We model stochastic transitions between compartments using the τ-leap method (*1*,*2*) with key parameters given in Appendix Table 2. Hospitalization parameters are shown in Appendix Table 3. Assuming that the events at each time-step are independent and do not impact the underlying transition rates, the numbers of each type of event should follow Poisson distributions with means equal to the rate parameters. We thus simulate the model according to the following equations:

$S_{a,r}(t+1) - S_{a,r}(t) = -P_1$

$E_{a,r}(t+1) - E_{a,r}(t) = P_1 - P_2$

$IaA,r\,(t+1) - IaA,r\,(t) = (1-\tau)P\,2 - P\,3$

$IaY,r\,(t+1) - IaY,r\,(t) = \tau P\,2 - P\,4 - P\,5$

$IaH,r\,(t+1) - IaH,r\,(t) = P\,5 - P\,6 - P\,7$

$R_{a,r}(t+1) - R_{a,r}(t) = P_3 + P_4 + P_6$

$D_{a,r}(t+1) - D_{a,r}(t) = P_7$,

with

$P_1 \sim Pois(S_{a,r}(t)F_{a,r}(t))$

$P_2 \sim Pois(\sigma E_{a,r}(t))$

$P_3 \sim Pois(\gamma^A I_a{}^A{}_{,r}(t))$

$P_4 \sim Pois((1-\pi)\gamma^Y I_a{}^Y{}_{,r}(t))$

$P_5 \sim Pois(\pi\eta I_a{}^Y{}_{,r}(t))$

$P_6 \sim Pois((1-\nu)\gamma^H I_a{}^H)$

$P_7 \sim Pois(\nu\mu I_{a,r}^{H}(t))$

and where $F_{a,r}$ denotes the force of infection for individuals in age group a and risk group r and is given by:

$$F_{a,r}(t) = \sum_{i \in A} \sum_{j \in K} \left(I_{i,j}^{Y}(t)\omega^{Y} + I_{i,j}^{A}(t)\omega^{A} + E_{i,j}(t)\omega^{E}\right) \beta_{a,i}\phi_{a,i}/N_i .$$

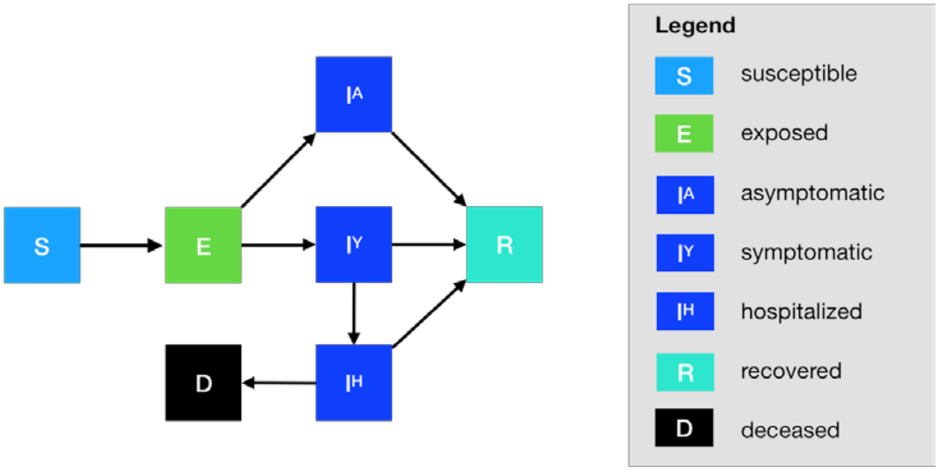

Appendix Figure 1. Compartmental model of COVID-19 transmission in a US city. Each subgroup **(**defined by age and risk) is modeled with a separate set of compartments. Upon infection, susceptible individuals (*S*) progress to exposed (*E*) and then to either symptomatic infectious (I^Y) or asymptomatic infectious (*IA*). All asymptomatic cases eventually progress to a recovered class where they remain protected from future infection (*R*); symptomatic cases are either hospitalized (*I*) or recover. Mortality (*D*) varies by age group and risk group and is assumed to be preceded by hospitalization.

Appendix Table 1. Initial conditions, school closures and social distancing policies

Variable	Settings
Initial day of simulation	3/1/2020
Initial infection number in locations	5 symptomatic cases in 18–49y age group
Trigger to close school	3/14/2020
Closure Duration	Until start of 2020–2021 school year (8/17/20)
α: Reduction of nonhousehold contacts (work and other)	Five scenarios: 0, 0.25, 0.5, 0.75, 0.9
Age-specific and day-specific contact rates	Home, work, other and school matrices provided in Appendix Tables 4–7; Normal weekday = home + work + other + school; Normal weekend = home + other; Normal weekday holiday = home + other; Normal weekday during summer or winter break = home + work + other; School closure weekday = home + (1 – α) × (work + other); School closure weekend = home + (1 – α) × (other); School closure weekday holiday = home + (1 – α) × (other); School closure during summer or winter break = home + (1 – α) × (work + other)

Appendix Table 2. Model parameters in which values that are given as 5element vectors are age-stratified with values corresponding to 0–4, 5–17, 18–49, 50–64, 65+ year age groups, respectively*

Parameter	Best guess – values (doubling time = 7.2 d)	Best guess values (doubling time = 4 d)	Source
R0	2.2	2.2	Li et al. (*3*)
δ: doubling time	7.2 d	4 d	Kraemer et al. (*4*)
β: transmission rate*	0.0311915	0.0500845	Fitted[a] to obtain specified *R0* given δ
γ^A : recovery rate on asymptomatic infectious compartment	Equal to γ^Y		
γ^Y: recovery rate on symptomatic infectious nontreated compartment	$\frac{1}{Y}$ ~ *Triangular* (5.3, 6.3, 7.3) γ		(*5*)
τ: symptomatic proportion (%)	57		(*6*)
σ: exposed rate	$\frac{1}{}$~ *Triangular* (1.9, 2.9, 3.9) σ		Based on incubation (*7*) and presymptomatic periods (*5*)
ω^E: relative infectiousness of individuals in compartment E	$\omega^E = 0$		
ω^A : relative infectiousness of infectious individuals in compartment I^A	2 3		He et al. (*8*)
IFR: infected fatality ratio, age specific (%)	Overall: [0.0016, 0.0049, 0.084, 1.000, 3.371]; Low risk: [0.00091668, 0.0021789, 0.03388, 0.25197, 0.64402]; High risk: [0.009167, 0.02179, 0.33878, 2.5197, 6.4402]		Age adjusted from Verity et al. (*9*)
YFR: symptomatic fatality ratio, age specific (%)	Overall: [0.002807, 0.008678, 0.1479, 1.755, 5.915]; Low risk: [0.001608, 0.003823, 0.05943,0.4420, 1.130]; High risk: [0.01608, 0.03823, 0.5943, 4.420, 11.30]		*Y FR* = *IF*τ*R*
h: high-risk proportion, age specific (%)	[8.2825, 14.1121, 16.5298, 32.9912, 47.0568]		Estimated using 2015–2016; Behavioral Risk Factor; Surveillance System (BRFSS) data with multilevel regression and poststratification using CDC's list of conditions that might increase the risk of serious complications from influenza (*10*–*12*)
rr: relative risk for high-risk persons compared with low risk in their age group	10		Assumption
School calendars	Austin Independent School District calendar (2019–2020, 2020–2021)		(*13*)

*The parameter β is fitted through constrained trust-region optimization in SciPy/Python (14). Given a value of β, a deterministic simulation is run based on central values for each parameter, from which we can compute the implied R_0 (β). We (*1*) track the daily number of new cases I_t (both symptomatic and asymptomatic) during the exponential growth portion of the epidemic (*2*), compute the log of the number of new cases: $y_t = log\ (I_t)$ and (*3*) use least squares to fit a line to this curve: $log\ (I_t) = y_0 + g \times t$. We then estimate the reproduction number R_0 (β) of the simulation for that specific value of β as R_0 (β) = 1 Γ + g × 1 where Γ is the generation time given by Γ = δ(R_0 – 1)/log(2). The optimizing function runs until the resulting value of R_0 (β) does not get closer to the target value.

We (*1*) track the daily number of new cases I_t (both symptomatic and asymptomatic) during the exponential growth portion of the epidemic (*2*), compute the log of the number of new cases: $y_t = log\ (I_t)$ and (*3*) use least squares to fit a line to this curve: $log\ (I_t) = y_0 + g \cdot t$. We then estimate the reproduction number R_0 (β) of the $\delta(R^0 - 1)$ simulation for that specific value of β as $R_0(\beta) = \Gamma \cdot g + 1$ where Γis the generation time given by $\Gamma = \frac{}{log\ (2)}$.

The optimizing function runs until the resulting value of R_0 (β) does not get closer to the target value.

Appendix Table 3. Hospitalization parameters

Parameter	Value	Source
γ^H : recovery rate in hospitalized compartment	0.0912409	10.96 d-average from admission to discharge (Fit to Austin admissions and discharge data)
YHR: symptomatic case hospitalization rate (%)	Overall: [0.07018, 0.07018, 4.735, 16.33, 25.54]; Low risk: [0.04021, 0.03091, 1.903, 4.114, 4.879]; High risk: [0.4021, 0.3091, 19.03, 41.14, 48.79]	Age adjusted from Verity et al. (9)
π: rate of symptomatic individuals go to hospital, age-specific	$\pi = \frac{\gamma^Y \cdot YHR}{\eta+(\gamma^Y-\eta)YHR}$	
η: rate from symptom onset to hospitalized	0.12195	5.9 d average from symptom onset to hospital admission (L. Tindale et al., unpub. data, https://doi.org/10.1101/2020.03.03.20029983) and 2.3 d pre-symptomatic period from He et al. (5)
μ: rate from hospitalized to death	0.12821	7.8 d-average from admission to death (Fit to Austin admissions and discharge data)
HFR: hospitalized fatality ratio, age specific (%)	[4, 12.365, 3.122, 10.745, 23.158]	$HFR = \frac{YFR}{YHR}$
ν: death rate on hospitalized individuals age specific	[0.0390, 0.1208, 0.0304, 0.1049, 0.2269]	$\nu = \frac{\gamma^H HFR}{\mu+(\gamma^H-\mu)HFR}$
ICU: proportion hospitalized people in ICU	[0.15, 0.20, 0.15, 0.20, 0.15]	CDC COVID-19 planning scenarios (based on US seasonal flu data)
Vent: proportion of individuals in ICU needing ventilation	[0.35, 0.3, 0.45, 0.5, 0.45]	CDC planning scenarios (based on US seasonal flu data)
d_{ICU} : duration of stay in ICU	8 d	Assumption, computed as average of hospital stay and ventilation durations
d_V : duration of ventilation	5 d	CDC COVID-19 planning scenarios
HCS:healthcare capacity	Hospital bed: 4,299; ICU bed: 755; Ventilator: 755	Estimates provided by each of the region's hospital systems and aggregated by regional public health leaders

Appendix Table 4. Home contact matrix (daily number contacts by age group at home)

Age, y	0–4	5–17	18–49	50–64	≥65
<1–4	0.5	0.9	2.0	0.1	0.0
5–17	0.2	1.7	1.9	0.2	0.0
18–49	0.2	0.9	1.7	0.2	0.0
50–64	0.2	0.7	1.2	1.0	0.1
≥65	0.1	0.7	1.0	0.3	0.6

Appendix Table 5. School contact matrix (daily number contacts by age group at school)

Age, y	0–4	5–17	18–49	50–64	≥65
<1–4	1.0	0.5	0.4	0.1	0.0
5–17	0.2	3.7	0.9	0.1	0.0
18–49	0.0	0.7	0.8	0.0	0.0
50–64	0.1	0.8	0.5	0.1	0.0
≥65	0.0	0.0	0.1	0.0	0.0

Appendix Table 6. Work contact matrix (daily number contacts by age group at work)

Age, y	0–4	5–17	18–49	50–64	≥65
>1–4	0.0	0.0	0.0	0.0	0.0
5–17	0.0	0.1	0.4	0.0	0.0
18–49	0.0	0.2	4.5	0.8	0.0
50–64	0.0	0.1	2.8	0.9	0.0
≥65	0.0	0.0	0.1	0.0	0.0

Appendix Table 7. Others contact matrix (daily number contacts by age group at other locations)

Age, y	0–4	5–17	18–49	50–64	≥65
>1–4	0.7	0.7	1.8	0.6	0.3
5–17	0.2	2.6	2.1	0.4	0.2
18–49	0.1	0.7	3.3	0.6	0.2
50–64	0.1	0.3	2.2	1.1	0.4
≥65	0.0	0.2	1.3	0.8	0.6

Section 2. Estimation of age-stratified proportion of population at high risk for COVID-19 complications

High-risk conditions for influenza and data sources for prevalence estimation are shown in Appendix Table 8. We estimate age-specific proportions of the population at high risk of complications from COVID-19 based on data for Austin, TX and Round-Rock, TX from the CDC's 500 cities project (Appendix Figure 2) (*15*). We assume that high risk conditions for COVID-19 are the same as those specified for influenza by the CDC (*10*). The CDC's 500 cities project provides city-specific estimates of prevalence for several of these conditions among adults (*16*). The estimates were obtained from the 2015–2016 Behavioral Risk Factor Surveillance System (BRFSS) data using a small-area estimation method known as multilevel regression and poststratification (*11*,*12*). It links geocoded health surveys to high spatial resolution population demographic and socioeconomic data (*12*).

Projected weekly incident COVID-19 cases are shown in Appendix Figure 3, and projected COVID-19 healthcare demand and cumulative deaths are shown in Appendix Figure 4.

Estimating High-Risk Proportions for Adults

To estimate the proportion of adults at high risk for complications, we use the CDC's 500 cities data, as well as data on the prevalence of HIV/AIDS, obesity and pregnancy among adults (Appendix Table 2).

The CDC 500 cities dataset includes the prevalence of each condition on its own, rather than the prevalence of multiple conditions (e.g., dyads or triads). Thus, we use separate co-morbidity estimates to determine overlap. Reference about chronic conditions (*17*) gives US estimates for the proportion of the adult population with 0, 1 or $\geq$2 chronic conditions, per age group. Using this and the 500 cities data we can estimate the proportion of the population p_{HR} in each age group in each city with $\geq$1 chronic condition listed in the CDC 500 cities data (Appendix Table 2) putting them at high risk for flu complications.

HIV

We use the data from Table 20 in a CDC HIV surveillance report (*18*) to estimate the population in each risk group living with HIV in the U.S. (last column, 2015 data). Assuming independence between HIV and other chronic conditions, we increase the proportion of the

population at high-risk for influenza to account for individuals with HIV but no other underlying conditions.

Morbid Obesity

A BMI >40 kg/m^2 indicates morbid obesity and is considered high risk for influenza. The 500 Cities Project reports the prevalence of obese people in each city with BMI > 30 kg/m^2 (not necessarily morbid obesity). We use the data from Table 1 in Sturm and Hattori (*19*) to estimate the proportion of people with a BMI >30 that actually have a BMI >40 (across the United States); we then apply this to the 500 Cities obesity data to estimate the proportion of people who are morbidly obese in each city. Table 1 of Morgan et al. (*20*) suggests that 51.2% of morbidly obese adults have $\geq$1 other high risk chronic condition, and update our high-risk population estimates accordingly to account for overlap.

Pregnancy

We separately estimate the number of pregnant women in each age group and each city, following the methods in the CDC reproductive health report (*21*). We assume independence between any of the high-risk factors and pregnancy, and further assume that half the population are women.

Estimating High-Risk Proportions for Children

Since the 500 Cities Project only reports data for adults $\geq$18 years of age, we take a different approach to estimating the proportion of children at high risk for severe influenza. The 2 most prevalent risk factors for children are asthma and obesity; we also account for childhood diabetes, HIV and cancer.

From Miller et al. (*22*), we obtain national estimates of chronic conditions in children. For asthma, we assume that variation among cities will be similar for children and adults. Thus, we use the relative prevalence of asthma in adults to scale our estimates for children in each city. The prevalence of HIV and cancer in children are taken from CDC HIV surveillance report (*18*) and cancer research report (*23*), respectively.

We first estimate the proportion of children having either asthma, diabetes, cancer or HIV (assuming no overlap in these conditions). We estimate city-level morbid obesity in children using the estimated morbid obesity in adults multiplied by a national constant ratio for each age group estimated from Hales et al. (*24*), this ratio represents the prevalence in morbid obesity in children given the one observed in adults. From Morgan et al. (*20*), we estimate that 25% of morbidly obese children have another high-risk condition and adjust our final estimates accordingly.

Resulting Estimates

We compare our estimates for the Austin-Round Rock Metropolitan Area to published national-level estimates (*25*) of the proportion of each age group with underlying high risk conditions (Appendix Table 9). The biggest difference is observed in older adults, with Austin having a lower proportion at risk for complications for COVID-19 than the national average; for 25–39 year-old the high risk proportion is slightly higher than the national average.

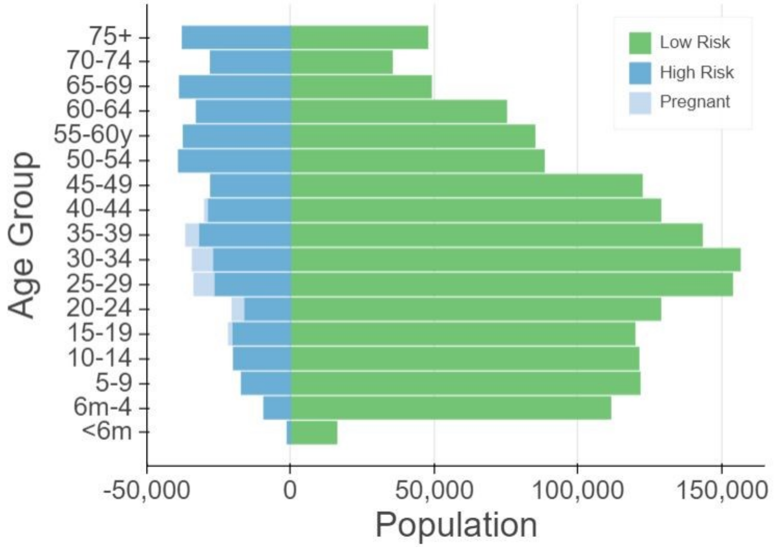

Appendix Figure 2. Demographic and risk composition of the Austin-Round Rock population. Bars indicate age-specific population sizes, separated by low risk, high risk, and pregnant. High risk is defined as individuals with cancer, chronic kidney disease, COPD, heart disease, stroke, asthma, diabetes, HIV/AIDS, and morbid obesity, as estimated from the CDC 500 Cities Project (*15*), reported HIV prevalence (*18*) and reported morbid obesity prevalence (*19*,*20*), corrected for multiple conditions. The population of pregnant women is derived using the CDC's method combining fertility, abortion and fetal loss rates (*26–28*).

Appendix Table 8. High-risk conditions for influenza and data sources for prevalence estimation

Condition	Data source
Cancer (except skin), chronic kidney disease, COPD, coronary heart disease, stroke, asthma, diabetes	CDC 500 cities (*29*)
HIV/AIDS	CDC HIV Surveillance report (*30*)
Obesity	CDC 500 cities (*29*), Sturm and Hattori (*19*), Morgan et al. (*20*)
Pregnancy	National Vital Statistics Reports (*31*) and abortion data (*27*)

Appendix Table 9. Comparison between published national estimates and Austin-Round Rock MSA estimates of the percent of the population at high-risk of influenza/COVID-19 complications

Age group	National estimates (24)	Austin (excluding pregnancy)	Pregnant women (proportion of age group)
<1 to 6 mo	NA	6.8	–
6 mo to 4 y	6.8	7.4	–
5 to 9 y	11.7	11.6	–
10 to 14 y	11.7	13.0	–
15 to 19 y	11.8	13.3	1.7
20 to 24 y	12.4	10.3	5.1
25 to 34 y	15.7	13.5	7.8
35 to 39 y	15.7	17.0	5.1
40 to 44 y	15.7	17.4	1.2
45 to 49 y	15.7	17.7	–
50 to 54 y	30.6	29.6	–
55 to 60 y	30.6	29.5	–
60 to 64 y	30.6	29.3	–
65 to 69 y	47.0	42.2	–
70 to 74 y	47.0	42.2	–
≥75 y	47.0	42.2	–

Section 3. Sensitivity Analysis with Respect to R_0

Our base scenarios assume a basic reproductive number (*R0*) of 2.2. Here, we provide projections assuming that COVID-19 has a higher reproduction number of *R0* = 3.5, with a doubling time of both 4 days and 7.2 days. Assuming this faster transmission scenario, higher levels of social distancing are required to reduce the burden of the disease.

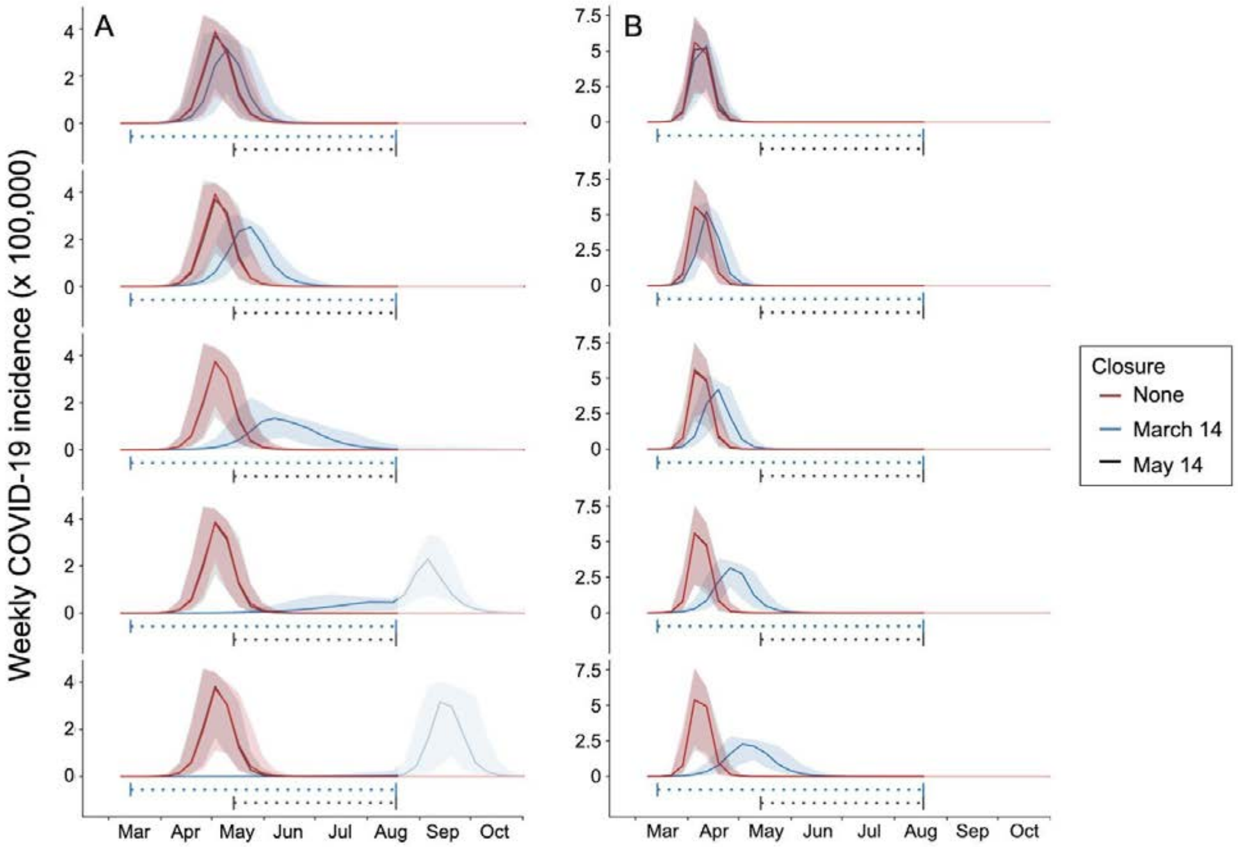

Appendix Figure 3. Projected weekly incident COVID-19 cases in the Austin-Round Rock MSA. Graphs show simulation results for different levels of social distancing and implementation times, assuming R_0 = 3.5 and an epidemic doubling time of A) 7.2 days (*19–22*) or B) 4 days (*22*,*24*,*25*). Each graph displays 3 projections: a baseline assuming no social distancing (red), social distancing implemented March 14-Aug 17, 2020 (blue), and social distancing implemented May 14-Aug 17, 2020 (black). From top to bottom, the graphs in each column correspond to increasingly stringent social distancing measures: school closures plus social distancing that reduces nonhousehold contacts by 0%, 25%, 50%, 75%, or 90%. Solid lines indicate the median of 100 stochastic simulations; shading indicates the inner 95% range of values. The horizontal dotted lines beneath the curves indicate intervention periods. The faded mid-August to December time range indicates long-range uncertainty regarding COVID-19 transmission dynamics and intervention policies.

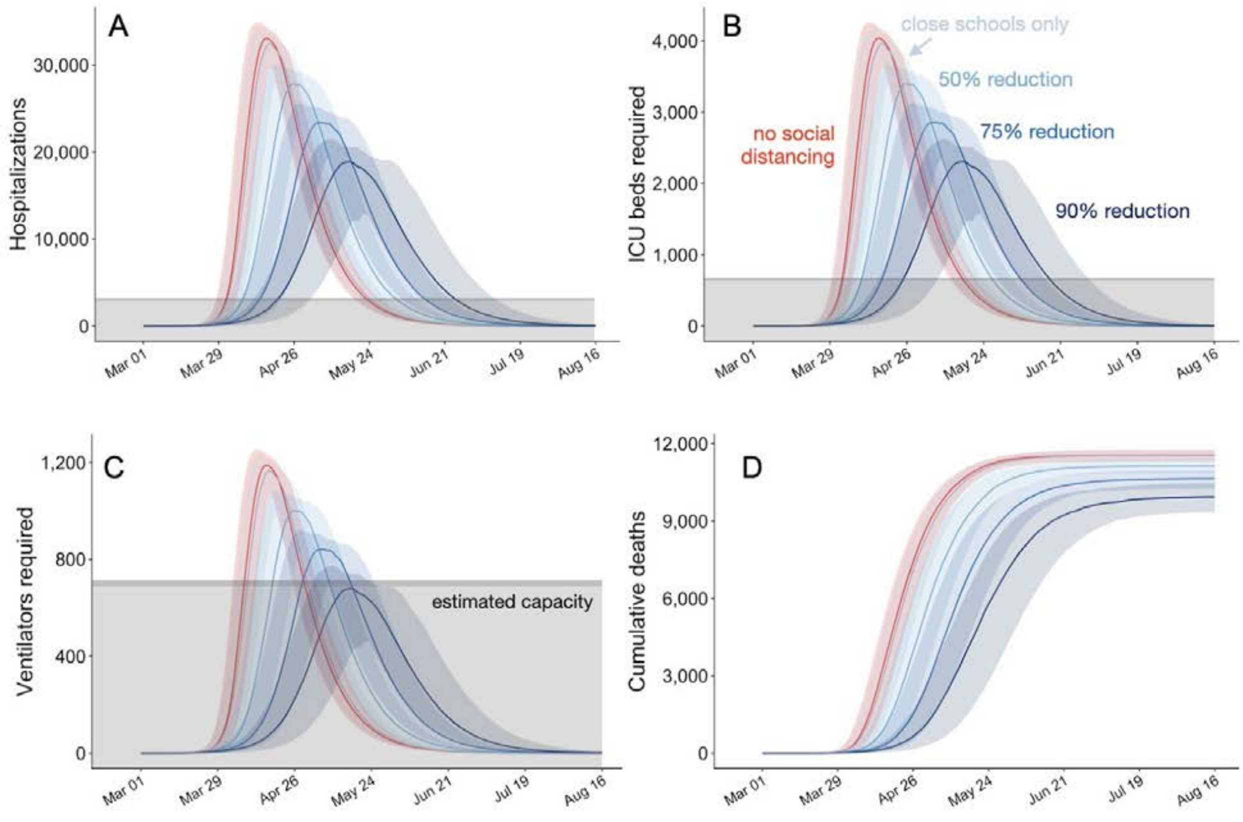

Appendix Figure 4. Projected COVID-19 healthcare demand and cumulative deaths in the Austin-Round Rock MSA from March 1 to August 17, 2020. Graphs show simulation results across multiple levels of social distancing, assuming *R* 0 = 3.5 with a 4-day epidemic doubling time. Extensive social distancing is expected to substantially reduce the burden of COVID-19 A) hospitalizations, B) patients requiring ICU care, C) patients requiring mechanical ventilation, and D) deaths. The red lines project COVID-19 transmission assuming no interventions under the parameters given in Table A1. The blue lines show increasing levels of social distancing interventions, from light to dark: school closures plus social distancing interventions that reduce nonhousehold contacts by either 0%, 50%, 75% or 90%. Lines and shading indicate the median and inner 95% range of values across 100 stochastic simulations. Gray shaded region indicates estimated surge capacity for COVID-19 patients in the Austin-Round Rock MSA as of March 28, 2020, which is calculated based on 80% of 42,99 hospital beds and 90% of 755 ICU beds and 755 mechanical ventilators.

Section 4. Sensitivity Analysis with Respect to Healthcare Durations

With the assumption that the healthcare system is likely to perform less effectively under the highly stressed condition, patient discharge might take longer in the surge setting. As sensitivity analysis, we analyzed longer duration hospital, ICU and ventilator treatment (Appendix Table 10). The results are summarized in Appendix Tables 11, 12 and Appendix Figure 5.

Appendix Table 10. Updated hospitalization parameters for which all values were modified based on discussions with Austin-Round Rock Medical authorities regarding worst case surge scenarios

Parameters	Original	Updated for sensitivity analysis	Details
γ^H: recovery rate in hospitalized compartment	0.0869565	0.07142857	14 d average from admission to discharge
μ: rate from hospitalized to death	0.0892857	0.07142857	14 d average from admission to death
Vent: proportion of individuals in ICU needing ventilation	0.35, 0.3, 0.45, 0.5, 0.45	0.67 (all ages)	
d_{ICU} : duration of stay in ICU	8 d	14 d	
d_V : duration of ventilation	5 d	10 d	

Appendix Table 11. Longer treatment surge scenario: estimated cumulative COVID-19 cases, healthcare requirements and deaths. The values are medians (with 95% prediction interval in parentheses) across 100 stochastic simulations for the Austin-Round Rock MSA from March 1 through August 17, 2020 based on the parameters given in Appendix Table 10

Outcome	No measures	School closure	School closure and 50% social distancing	School closure and 75% social distancing	School closure and 90% social distancing
Hospitalizations	79,788 (75,891-82,399)	76,873 (71,552-80,870)	40,719 (17,031-57,014)	2,120 (148-9,939)	118 (14-546)
ICU	13,415 (12,775-13,859)	12,919 (12,025-3,587)	6,841 (2,859-9,581)	356 (25-1,673)	20 (2-92)
Ventilators	8,943(8,517-9,239)	8,612 (8,016-9,058)	4,561 (1,906-6,388)	237 (17-1,115)	13 (2-61)

Appendix Table 12. Longer treatment surge scenario: estimated peak COVID-19 healthcare demands. The values are medians (with 95% prediction interval in parentheses) across 100 stochastic simulations for the Austin-Round Rock MSA from March 1 through August 17, 2020 based on the parameters given in Appendix Table 10

Outcome	No measures	School closure	School closure and 50% social distancing	School closure and 75% social distancing	School closure and 90% social distancing
Hospitalizations	27,678 (25,651-29,286)	25,347 (21,806-27,588)	8,862 (5,164-12,492)	564 (30-2,953)	22 (4-117)
ICU	4,669 (4,323-4,944)	4,273 (3,673-4,649)	1,488 (868-2,101)	95 (5-496)	4 (1-20)
Ventilators	2,223 (2,059-2,354)	2,035 (1,749-2,214)	709 (413-1,000)	45 (2-236)	2 (0-9)

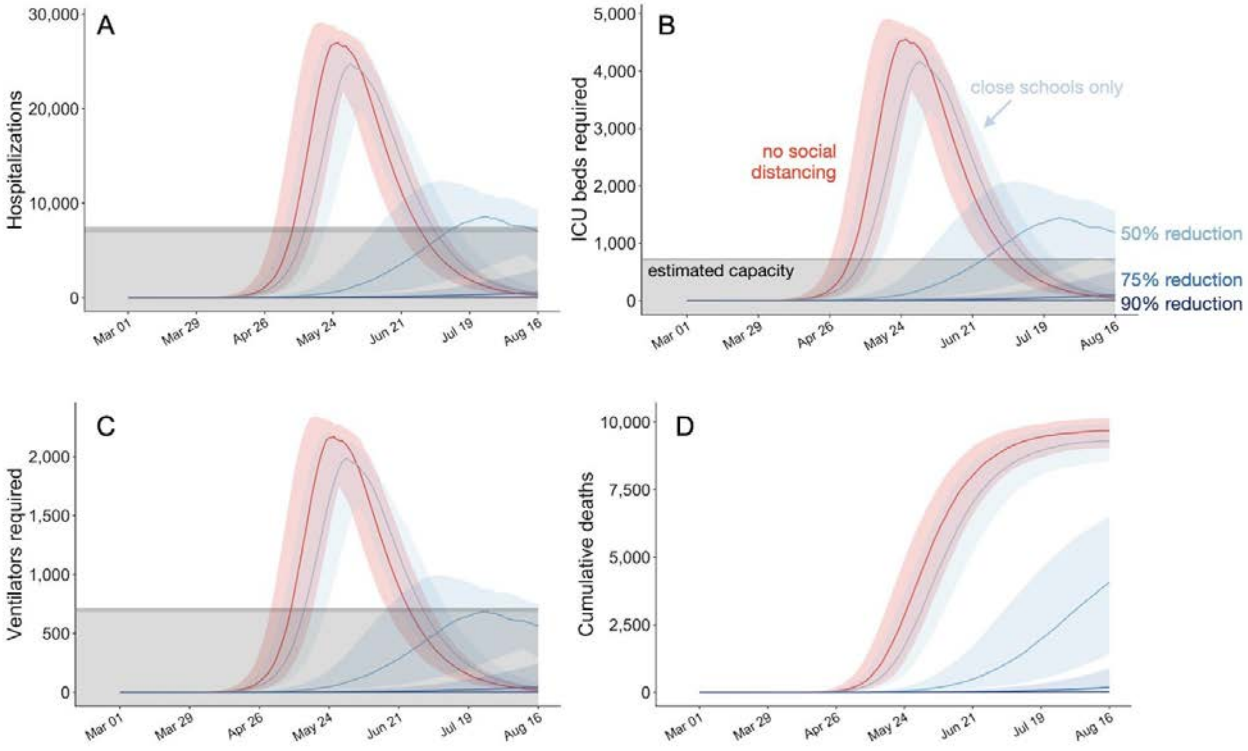

Appendix Figure 5. Longer treatment surge scenario: projected COVID-19 healthcare demand and cumulative deaths in the Austin-Round Rock MSA from March 1 to August 17, 2020. Graphs show simulation results across multiple levels of social distancing, assuming *R* 0 = 2.2 with a 4-day epidemic doubling time. Extensive social distancing is expected to substantially reduce the burden of COVID-19 A) hospitalizations, B) patients requiring ICU care, C) patients requiring mechanical ventilation, and D) deaths. The red lines project COVID-19 transmission assuming no interventions under the parameters given in Table A1. The blue lines show increasing levels of social distancing interventions, from light to dark: school closures plus social distancing interventions that reduce nonhousehold contacts by either 0%, 50%, 75% or 90%. Lines and shading indicate the median and inner 95% range of values across 100 stochastic simulations. Gray shaded region indicates estimated surge capacity for COVID-19 patients in the Austin-Round Rock MSA as of March 28, 2020, which is calculated based on 80% of 4,299 hospital beds and 90% of 755 ICU beds and 755 mechanical ventilators.

Section 5. Impact of 2-Week and 4-Week Delays in Implementation of Social Distancing Interventions, 2020.

We also modeled intermediate delays of 2 weeks (March 28) and 4 weeks (April 11). Even 2-week delays undermine the efficacy of the interventions with respect to reducing healthcare demand below local capacity (Appendix Figure 6, Appendix Table 13).

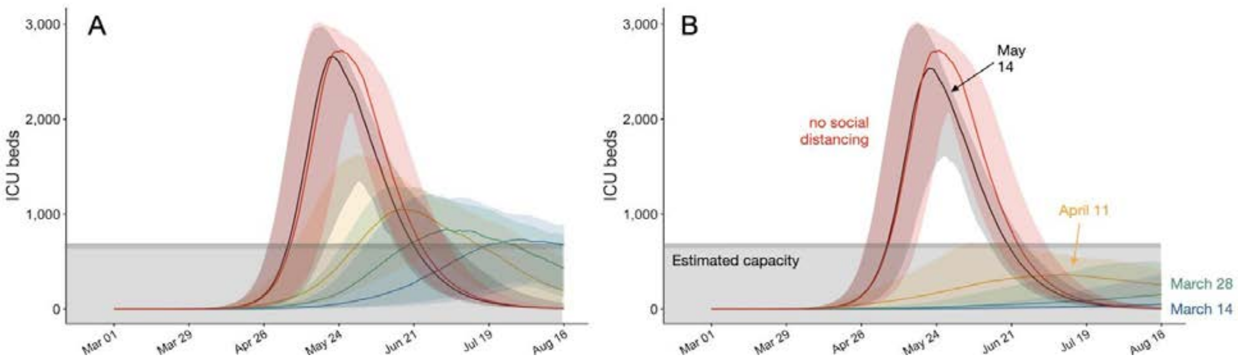

Appendix Figure 6. Graphs show simulation results for school closures with A) 50% reduction in nonhousehold contacts and B) 75% reduction in nonhousehold contacts, assuming *R* 0 = 2.2 with a 4-day epidemic doubling time. The red lines project COVID-19 transmission assuming no interventions under the parameters given in Appendix Table 1. The other lines colors indicate different delays in the timing of intervention: blue, green, yellow and black correspond to March 14, March 28, April 11, and May 14, 2020, respectively. Lines and shading indicate the median and inner 95% range of values across 100 stochastic simulations. Gray shaded region indicates estimated surge capacity for COVID-19 patients in the Austin-Round Rock MSA as of March 28, 2020, which is calculated based on 90% of 755 ICU beds.

Appendix Table 13. Date when COVID-19 healthcare requirements exceed capacity based on implementation date for school closures with 50% or 75% social distancing. Each value is a median across 100 stochastic simulations for the Austin-Round Rock MSA before August 17, 2020, based on the parameters given in Appendix Table 1

	School closure and 50% social distancing			School closure and 75% social distancing		
Outcome	March 14 start	March 28 start	April 11 start	March 14 start	March 28 start	April 11 start
Hospitalizations	July 1	June 7	May 23	Not exceed	Not exceed	Not exceed
ICU	July 20	June 20	May 31	Not exceed	Not exceed	Not exceed

References

1. Keeling MJ, Rohani P. Modeling infectious diseases in humans and animals. Princeton (NJ): Princeton University Press; 2011.

2. Gillespie DT. Approximate accelerated stochastic simulation of chemically reacting systems. J Chem Phys. 2001;115:1716–33. https://doi.org/10.1063/1.1378322

3. Li Q, Guan X, Wu P, Wang X, Zhou L, Tong Y, et al. Early transmission dynamics in Wuhan, China, of novel coronavirus-infected pneumonia. N Engl J Med. 2020;382:1199–207. PubMed https://doi.org/10.1056/NEJMoa2001316

4. Kraemer MU, Yang C-H, Gutierrez B, Wu C-H, Klein B, Pigott DM, et al.; Open COVID-19 Data Working Group. The effect of human mobility and control measures on the COVID-19 epidemic in China. Science. 2020;368:493–7. PubMed https://doi.org/10.1126/science.abb4218

5. He X, Lau EH, Wu P, Deng X, Wang J, Hao X, et al. Temporal dynamics in viral shedding and transmissibility of COVID-19. Nat Med. 2020;26:672–5. PubMed https://doi.org/10.1038/s41591-020-0869-5

6. Gudbjartsson DF, Helgason A, Jonsson H, Magnusson OT, Melsted P, Norddahl GL, et al. Spread of SARS-CoV-2 in the Icelandic population. N Engl J Med. 2020;382:2302–15. PubMed https://doi.org/10.1056/NEJMoa2006100

7. Zhang J, Litvinova M, Wang W, Wang Y, Deng X, Chen X, et al. Evolving epidemiology and transmission dynamics of coronavirus disease 2019 outside Hubei province, China: a descriptive and modelling study. Lancet Infect Dis. 2020;20:793–802. PubMed https://doi.org/10.1016/S1473-3099(20)30230-9

8. He D, Zhao S, Lin Q, Zhuang Z, Cao P, Wang MH, et al. The relative transmissibility of asymptomatic COVID-19 infections among close contacts. Int J Infect Dis. 2020;94:145–7. PubMed https://doi.org/10.1016/j.ijid.2020.04.034

9. Verity R, Okell LC, Dorigatti I, Winskill P, Whittaker C, Imai N, et al. Estimates of the severity of coronavirus disease 2019: a model-based analysis. Lancet Infect Dis. 2020;20:669–77. PubMed https://doi.org/10.1016/S1473-3099(20)30243-7

10. Centers for Disease Control and Prevention. People at high risk of flu, 2019 [cited 2020 Mar 26]. https://www.cdc.gov/flu/highrisk/index.htm

11. Centers for Disease Control and Prevention. Behavioral risk factor surveillance system, 2019 [cited 2020 Mar 26]. https://www.cdc.gov/brfss/index.html

12. Zhang X, Holt JB, Lu H, Wheaton AG, Ford ES, Greenlund KJ, et al. Multilevel regression and poststratification for small-area estimation of population health outcomes: a case study of chronic obstructive pulmonary disease prevalence using the behavioral risk factor surveillance system. Am J Epidemiol. 2014;179:1025–33. PubMed https://doi.org/10.1093/aje/kwu018

13. Austin ISD. Calendar of events [cited 2020 Mar 26]. https://www.austinisd.org/calendar

14. SciPy Community. minimize(method='trust-constr') — SciPy v1.4.1 Reference Guide [cited 2020 Mar 28]. https://docs.scipy.org/doc/scipy/reference/optimize.minimize-trustconstr.html

15. Centers for Disease Control and Prevention. 500 cities project: local data for better health, 2019 [cited 2020 Mar 19]. https://www.cdc.gov/500cities/index.htm

16. Centers for Disease Control and Prevention. Health outcomes: 500 cities, 2019 [cited 2020 Mar 28]. https://www.cdc.gov/500cities/definitions/health-outcomes.htm

17. PEW Research. Part one: who lives with chronic conditions, 2013 [cited 2019 Nov 23]. https://www.pewresearch.org/internet/2013/11/26/part-one-who-lives-with-chronic-conditions/

18. Centers for Disease Control and Prevention. HIV surveillance report, 2016; 2018 [cited 2020 Jul 15] https://www cdc gov/hiv/library/reports/hiv-surveil-lance html

19. Sturm R, Hattori A. Morbid obesity rates continue to rise rapidly in the United States. Int J Obes. 2013;37:889–91. PubMed https://doi.org/10.1038/ijo.2012.159

20. Morgan OW, Bramley A, Fowlkes A, Freedman DS, Taylor TH, Gargiullo P, et al. Morbid obesity as a risk factor for hospitalization and death due to 2009 pandemic influenza A(H1N1) disease. PLoS One. 2010;5:e9694. PubMed https://doi.org/10.1371/journal.pone.0009694

21. Centers for Disease Control and Prevention, Division of Reproductive Health. Estimating the number of pregnant women in a geographic area [cited 202 Jul 15]. https://www.cdc.gov/reproductivehealth/emergency/pdfs/PregnacyEstimatoBrochure508.pdf

22. Miller GF, Coffield E, Leroy Z, Wallin R. Prevalence and costs of five chronic conditions in children. J Sch Nurs. 2016;32:357–64. PubMed https://doi.org/10.1177/1059840516641190

23. American Cancer Society. Cancer facts and figures, 2014 [cited 2020 Mar 30]. https://www.cancer.org/research/cancer-facts-statistics/all-cancer-facts-figures/cancer-facts-figures-2 014.html

24. Hales CM, Fryar CD, Carroll MD, Freedman DS, Ogden CL. Trends in obesity and severe obesity prevalence in US youth and adults by sex and age, 2007–2008 to 2015. JAMA. 2018;319:1723–5. PubMed https://doi.org/10.1001/jama.2018.3060

25. Zimmerman RK, Lauderdale DS, Tan SM, Wagener DK. Prevalence of high-risk indications for influenza vaccine varies by age, race, and income. Vaccine. 2010;28:6470–7. PubMed https://doi.org/10.1016/j.vaccine.2010.07.037

26. Martin JA, Hamilton BE, Osterman MJK, Driscoll AK, Drake P. Births: final data for 2017. Natl Vital Stat Rep. 2018;67:1–50. PubMed

27. Jatlaoui TC, Boutot ME, Mandel MG, Whiteman MK, Ti A, Petersen E, et al. Abortion surveillance—United States, 2015. MMWR Surveill Summ. 2018;67:1–45. PubMed https://doi.org/10.15585/mmwr.ss6713a1

28. Ventura SJ, Curtin SC, Abma JC, Henshaw SK. Estimated pregnancy rates and rates of pregnancy outcomes for the United States, 1990-2008. Natl Vital Stat Rep. 2012;60:1–21. PubMed

29. Centers for Disease Control and Prevention. 500 cities project: local data for better health, 2019 [cited 2020 Jun 22]. https://www.cdc.gov/500cities/index.htm

30. Centers for Disease Control and Prevention. Health outcomes: 500 cities, 2019 [cited 2020 Jun 22]. https://www.cdc.gov/500cities/definitions/health-outcomes.htm

31. Martin JA, Hamilton BE, Osterman MJ, Driscoll AK, Drake P. Births: Final Data for 2017. Natl Vital Stat Rep. 2018;67:1–50. PubMed
